# Consequences of delayed surgical intervention of a displaced midshaft clavicle fracture: a case report

**DOI:** 10.1016/j.xrrt.2023.03.004

**Published:** 2023-04-07

**Authors:** Gabrielle K. Van Scoy, Kaveh R. Sajadi, Tim L. Uhl

**Affiliations:** a Department of Physical Therapy, University of Kentucky, Lexington, KY, USA; b Kentucky Bone and Joint Surgeons, Lexington, KY, USA

**Keywords:** Osteotomy, Neurological involvement, Pain, Sleep disturbance, Malunion, Scapular dyskinesis

The clavicle is one of the most common fracture sites in children and adolescents aged up to 16 years, with approximately 8.1% of all fractures in this population.^5^ Fractures of the clavicle have historically been treated conservatively in the adolescent population due to low nonunion rates, but union is not the only measure of recovery.^7^^,^^11^ The most common location of these fractures is in the middle third of the clavicle, accounting for approximately 90% of clavicle fractures in children and adolescents, and often occurring with some degree of displacement.^13^ Compared to adults, fewer comparative studies have been performed for children and adolescents contrasting operative vs. conservative treatment of this type of fracture, leading to continued controversy regarding this topic.^12^ Much of this controversy stems from the belief that the pediatric or adolescent clavicle has great remodeling potential, as the lateral physis of the clavicle does not close until about 19 years of age and the medial physis around 25 years of age.^9^ However, most of the longitudinal growth of the clavicle is complete by 12 years of age, and since the vast majority of clavicle fractures in children and adolescents occur in the midshaft, remodeling capabilities may be limited, especially in cases of displacement and shortening.^9^^,^^12^ Generally, operative management of the displaced midshaft clavicle fracture in children and adolescents is recommended for only special circumstances regardless of amount of displacement or translation of the fracture.^12^ These circumstances may include open fractures, ipsilateral humerus and scapula fractures, neurovascular injuries, and potential for compromised skin integrity.^9^

Limited and conflicting evidence exists regarding treatment for the adolescent population.^4^^,^^9^^,^^12^ Some studies have concluded that both intermediate and long-term outcomes are similar with both operative and nonoperative management of displaced midshaft clavicle fractures in adolescents.^9^^,^^12^ One study noted that cases of fracture shortening were associated with lower Oxford Shoulder Scores as well as overall satisfaction in the outcome following 4.7 years after injury.^9^ Operative management of displaced midshaft clavicle fractures in patients aged 12 to 18 years reported faster functional recovery in addition to mildly improved radiological and functional outcomes compared to those treated nonoperatively.^4^ The authors concluded that overall, primary operative management of these types of fractures in adolescents ultimately led to less shoulder pain, less functional deficits, and ultimately better results compared to conservative management.^4^ Current best practice uses a patient-specific approach, as conservative or operative management are both appropriate treatment options. Factors to consider in the decision-making process include fracture pattern, comorbidities, and patient expectations.^6^

The purpose of this case report is to present the surgical management of an adult patient with a symptomatic clavicle malunion treated with a corrective osteotomy with internal fixation and bone allograft. This case report reinforces that radiographic union does not assure absence of long-term sequalae and return to full function following nonoperative management in an adolescent athlete.

## Case report

**Figure 1:**
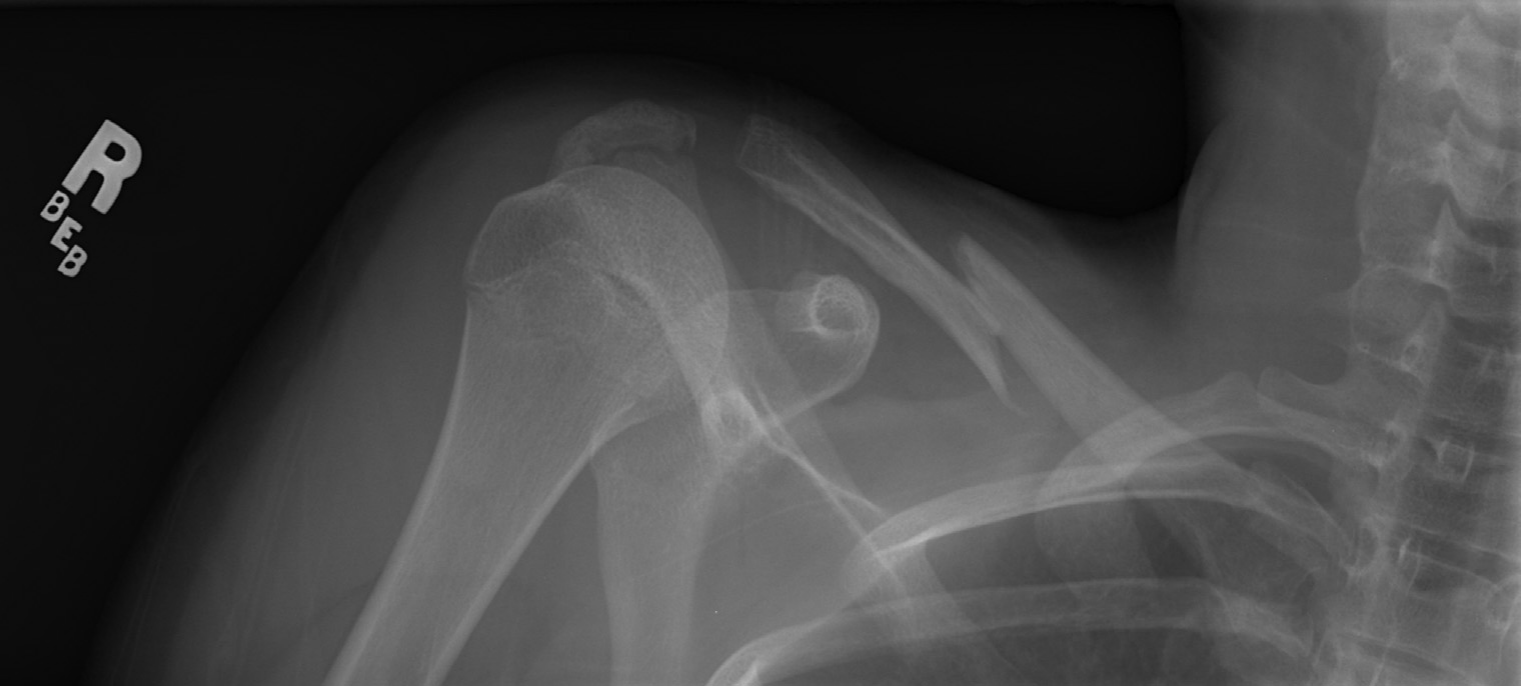
Anterior-posterior uptilt radiograph of original right displaced midshaft clavicle fracture of patient at 13 years old.

**Figure 2:**
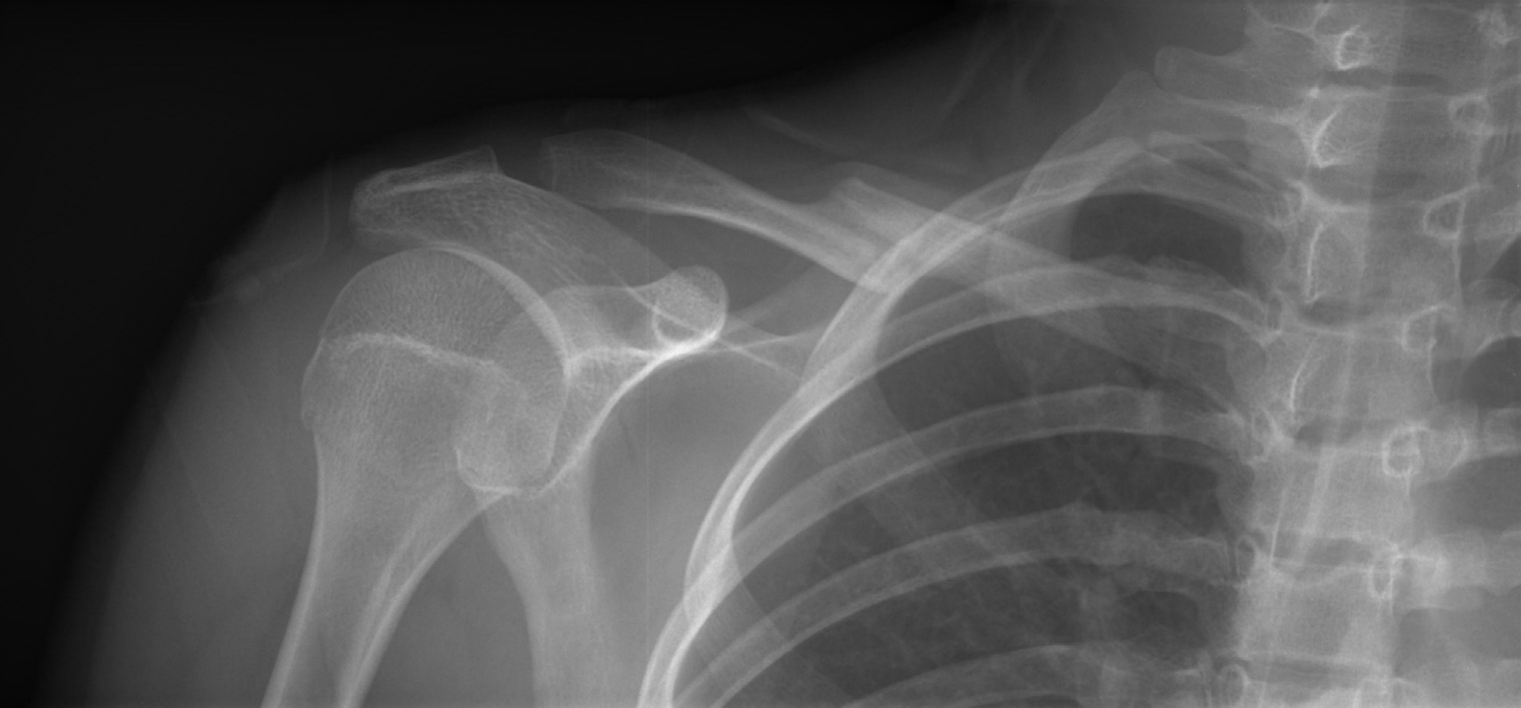
Anterior-posterior radiograph of right clavicle at last follow-up approximately 5 months following initial injury.

In late 2007, this 13-year-old female equestrian athlete fell from a horse and was taken to the emergency department where radiographs revealed a Robinson Type 2b1 fracture of the right clavicle (Fig. 1).^10^ Upon referral to a pediatric orthopedic surgeon, conservative management was chosen based solely on the patient’s age and historically low rates of nonunion of midshaft clavicle fractures in this population.^7^^,^^11^ This fracture was treated with a sling and swathe to immobilize the extremity. The patient reported tingling and discomfort in the right medial forearm within 1 week following the initial trauma, which was treated as a wrist sprain following negative wrist radiographs. Repeat radiographs revealed the fracture was slow to heal. The fracture ultimately achieved radiographic union following approximately 5 months (Fig. 2). During that time the patient stopped all activities until the fracture was healed and symptoms improved; however, parasthesias in the right medial forearm never completely resolved. At 6 months following initial injury, the patient was able to return to equestrian competition. However, over the next 13 years, this patient had intermittent nerve pain that caused aching in the medial forearm that was self-managed with over-the-counter anti-inflammatory medication. Residual right shoulder pain prevented the patient from sleeping on her right side for 13 years.

In June 2017, at 22 years old this same patient sustained a left midshaft clavicle fracture following a fall from a horse. This fracture was also treated conservatively with a sling, and the patient was able to return to horseback riding 6 weeks later; however, the fracture had not achieved complete radiographic union. Unfortunately, the patient sustained a second fracture of the midshaft of the left clavicle approximately 3 months later from a fall from a horse. This second fracture was managed surgically with open reduction internal fixation (ORIF) with an intramedullary nail, and the patient was able to return to equestrian competition 2 months later. The patient ultimately achieved radiographic and clinical union of the left clavicle fracture with no adverse events of the left upper extremity from that point forward.

In June 2020, at 25 years old the patient’s right medial forearm pain significantly worsened with an increase in equestrian training demand. The patient also experienced significant pain and weakness in the right shoulder and arm while transferring patients during her professional training as a physical therapy student. After 6 months of conservative self-management, symptoms remained unchanged, and she sought an orthopedic consult for her right shoulder and right medial forearm pain.

## Physical examination

**Figure 3:**
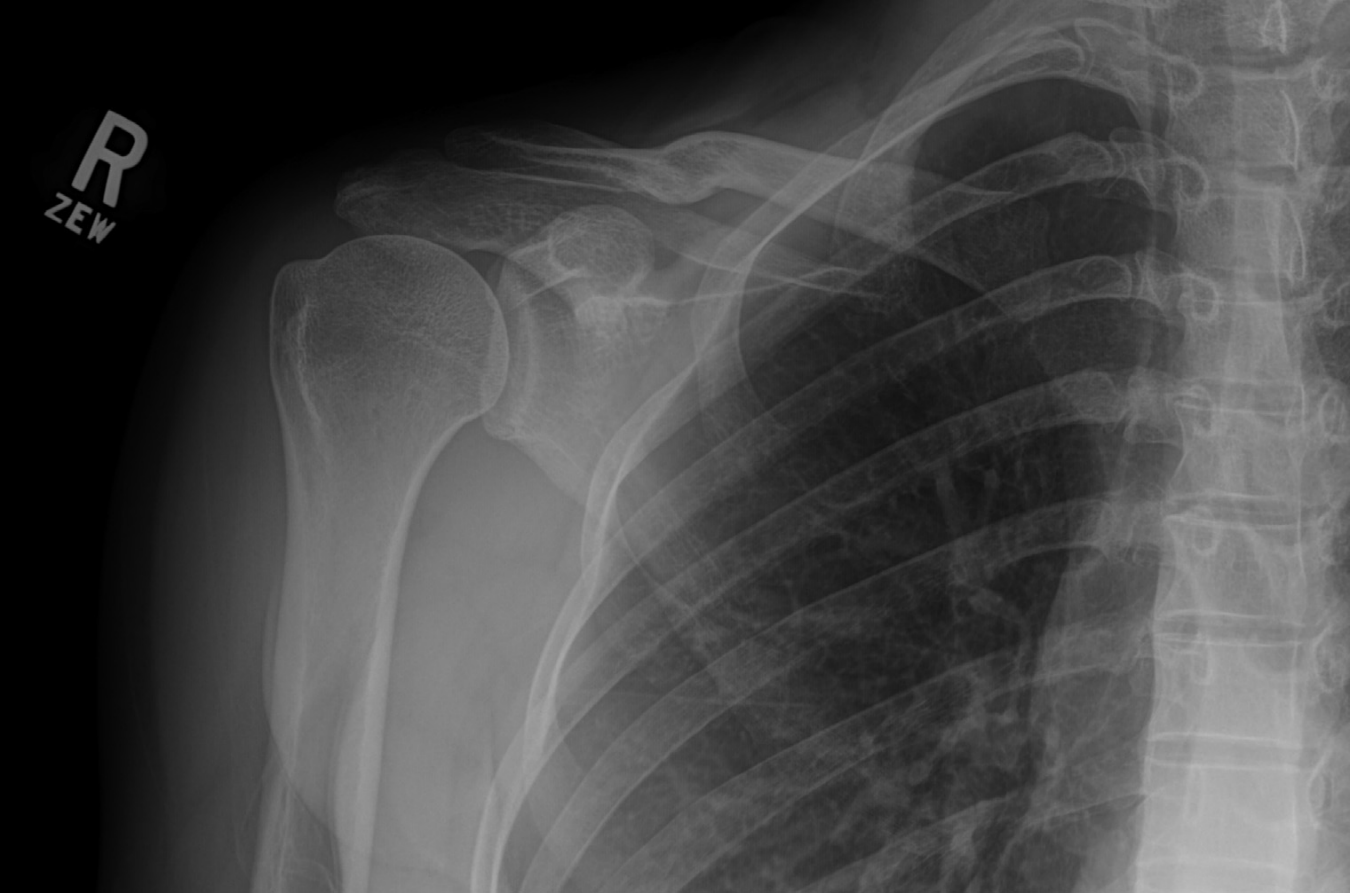
Preoperative axial radiograph of *Right* clavicle of patient at 26 years old.

The patient was a healthy 26-year-old female with no comorbidities and normal vital signs. Physical examination and systems review revealed her primary complaints were medial forearm pain and pain with resistance testing of all right shoulder motion. Observation during the physical examination revealed an obvious deformity of the right clavicle. Significant anterior tilt of the scapula at rest with mild scapular winging appreciated with both active flexion and abduction was noted and classified as scapular dyskinesis.^3^ Additionally, in resting posture, the right scapula was visibly elevated compared to the left. Scapular winging of the right shoulder was more pronounced with the hand behind the back (internal rotation functional reach). Physical examination of right shoulder active range of motion (AROM) was limited (Table I). Right shoulder isometric strength was slightly decreased compared to the left shoulder (Table II). The initial Quick Disabilities of the Arm, Shoulder, and Hand (QuickDASH) score was 22.7. Cervical active ROM was normal, Spurling’s test was negative, and Roos test was negative. The physical examination was inconclusive, so additional diagnostic imaging and testing were ordered (Fig. 3).

**Table I:** Preoperative and postoperative shoulder active range of motion (AROM).

	Preoperative		4 months postoperative		8 months postoperative	
	R	L	R	L	R	L
Flexion	142°	149°	150°	155°	174°	174°
Extension	46°	51°	53°	57°	50°	55°
Abduction	143°	162°	158°	166°	170°	179°
External Rotation	49°	64°	58°	58°	57°	60°
Internal Rotation Functional Reach	T7	T4	T5	T4	T3	T1

**Table II:** Preoperative and postoperative shoulder isometric strength (kg.).

	Preoperative		8 months postoperative	
	R	L	R	L
Flexion	8.8	10.4	9.2	9.0
External Rotation	12.9	12.8	13.4	13.4
Internal Rotation	14.0	16.1	14.7	13.8

Magnetic resonance imaging of the cervical spine was negative, and a nerve conduction study (NCS) and electromyographic testing of the right upper extremity revealed a medial antebrachial cutaneous neuropathy, ultimately leading to a diagnosis of thoracic outlet syndrome. The patient began formal physical therapy for treatment of thoracic outlet syndrome, in addition to receiving a corticosteroid injection of the pectoralis minor tendon. Three months later, the patient reported her right shoulder and medial forearm symptoms remained unchanged and the patient was referred for a second orthopedic consult. A computed tomography scan obtained of bilateral clavicles demonstrated approximately 15 mm of shortening. A corrective osteotomy was recommended to restore clavicular length, and the patient underwent a clavicle osteotomy with repair of malunion with internal fixation and bone allograft during a graduate school break.

## Surgical technique

**Figure 4:**
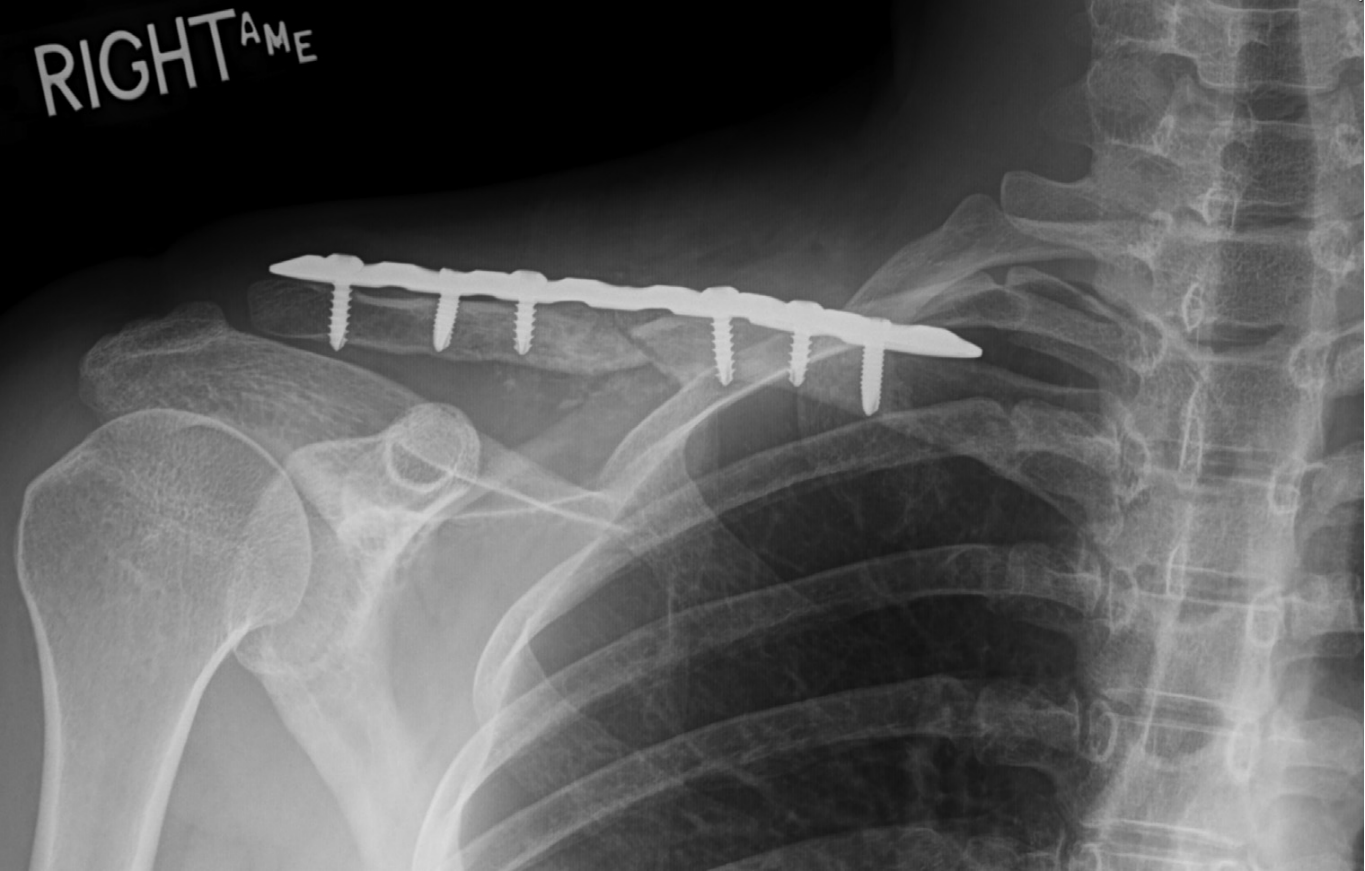
Postoperative anterior-posterior uptilt radiograph of *Right* clavicle.

Surgery was performed under general anesthesia and interscalene nerve block. The patient was placed in the beach chair position. A curvilinear incision was made along the clavicle centered over the malunion site and carried sharply through the skin and subcutaneous tissue. Flaps were developed over the platysma and trapezial pectoral fascia. The fascia was then divided directly over the clavicle and subperiosteal dissection used to expose the clavicle. The deformity could be appreciated with an apex posterior and superior direction. The osteotomy was planned and carried out in an oblique lateral to medial and superior to inferior direction. By mobilizing the lateral fragment to restore length and correct the apex posterior angulation, the scapula could be seen to correct into a normal alignment. Both edges of the osteotomy were opened with a drill to encourage healing. The fracture was appropriately fixed with the plate and screws. C arm fluoroscopy confirmed appropriate alignment of the fracture and placement of all hardware. Two bicortical cortical screws were placed on either side of the fracture and 1 locking screw on either side of the fracture. The osteotomy lengthened the clavicle by approximately 1 cm, which was felt to be the limits to allow overlap of fragments to minimize need for graft and risk of nonunion. The wound was irrigated, then 2 cc of vivigen bone graft from Synthes (West Chester, PA, USA) was packed into the fracture site and around the fracture site and plate, and routine wound closure was carried out (Fig. 4).

## Postoperative course

Postoperatively, the patient was placed in a simple sling for comfort only and permitted to begin active and passive ROM as tolerated. At approximately 1 week postoperatively, the patient developed superficial venous thromboses in the basilic vein of the arm and forearm, cephalic vein of the forearm, and median cubital vein. These were treated symptomatically with anti-inflammatories and warm compresses with complete resolution of symptoms within 2 months. The patient began physical therapy at 6 weeks with no limitations on active or passive ROM of the right shoulder, but no resistance work was permitted. At this time, the patient demonstrated improved right shoulder AROM (Table I) with no pain reported throughout the entire ROM. Mild to moderate right scapular winging persisted with both active shoulder flexion and abduction. At 10 weeks, the patient was permitted to begin resistance training with an emphasis on scapular strengthening. By 4 months postoperatively, the patient’s shoulder strength was improved and pain-free for all motions. After 6 months, the patient had a full asymptomatic return to all normal activities, including horseback riding and training for upcoming equestrian events without limitations. The patient’s final QuickDASH score was 0. A repeat NCS was performed of the right upper extremity at approximately 7 months postoperatively, which revealed complete resolution of the medial antebrachial cutaneous neuropathy. At 8 months of follow-up, the patient’s right shoulder AROM and isometric strength were comparable to the left shoulder (Table I, Table II and Table I, Table II).

## Discussion

This case report presents an athlete with a symptomatic clavicle malunion that suffered night pain, loss of function, and prolonged neurological symptoms for 13 years. Unfortunately, this may be more common than expected as Nowak et al,^8^ prospectively examined 208 patients aged 15 years or more who sustained a clavicle fracture and were treated conservatively. Ninety six (46%) of the patients had long-term sequelae at 9.5 years of follow-up similar to this patient.^8^ This suggests an earlier ORIF of this patient and other athletes may have prevented the need for corrective osteotomy and prevented negative long-term effects on quality of life this and other patients face.

Current management of midshaft clavicle fractures includes consideration of fracture pattern, comorbidities, and patient expectations to make case-by-case decisions regarding operative vs. conservative treatment.^6^ A recent prospective cohort study by Heyworth et al^1^ followed this approach without standardized intervention criteria. They concluded no difference in patients’ functional outcomes at 2 years between operative and nonoperative groups.^1^ This would suggest that the multiple surgeons involved in this multicenter study may have correctly chosen those patients who needed ORIF. However, 5%-7% of both groups had healing complications.^1^ It is relevant for physicians to consider in cases of athletes with continued symptoms and delayed healing that earlier surgical intervention may prevent long-term negative effects.

Earlier surgical management of this patient’s right displaced midshaft clavicle fracture may have produced better long-term outcomes. The symptoms of neuropathy within 1 week of injury were also not considered or addressed beyond wrist radiographs. This original fracture was slow to heal, requiring approximately 5 months of immobilization with a sling and swathe. At 6 months, the patient continued to have pain and weakness in the shoulder with activities, as well as being unable to sleep on the affected side. Additionally, although the aching in the medial forearm was no longer constant, it persisted intermittently with the same intensity. Perhaps further consideration of more aggressive treatment of these fractures should be given after 3 months of follow-up in the presence of a slow healing rate in addition to the presence of remaining sequalae. This case also suggests that an athlete with this type of fracture and related symptoms may not do well nonoperatively in long term.

In patients presenting with shoulder pain or neurological symptoms related to a symptomatic clavicle malunion, a corrective osteotomy with plate and screw fixation may offer relief of symptoms. Corrective osteotomies have been used successfully in the treatment of symptomatic malunions of midshaft clavicle fractures.^2^ In a retrospective study of 10 patients who were treated with a corrective osteotomy with plate and screw fixation, there was a significant improvement in the DASH score at a mean of 37 ± 17 months of follow-up.^2^ Preoperatively, the mean DASH score was 78 ± 14.9, and postoperatively the mean DASH score was 45 ± 23.2.^2^ Prior to surgery, the most common complaints of these patients were shoulder pain as well as arm weakness.^2^ The indication for corrective osteotomy was 1.5 cm or more clavicular shortening on a bilateral PA shoulder radiograph as well as finding no other explanation for the patient’s shoulder pain.^2^

**Figure 5:**
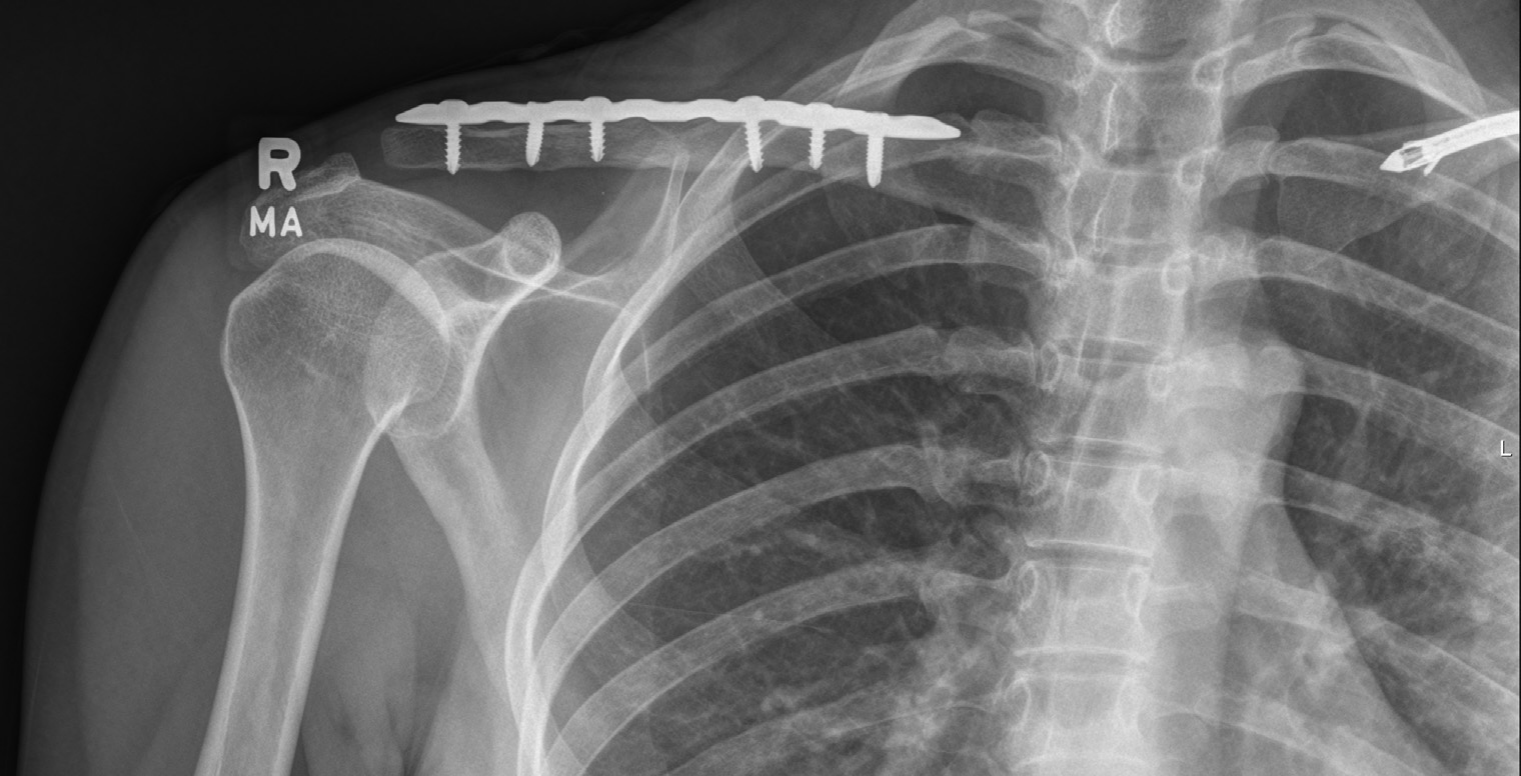
Axial radiograph of *Right* clavicle at 11 months postoperatively.

In this current case, the patient reports no symptoms with equestrian training or competition and has been able to sleep on the affected side for the first time since prior to the initial injury 13 years ago. Additionally, the patient reported complete resolution of aching in the medial forearm, and an NCS performed approximately 7 months postoperatively was a normal study of bilateral medial antebrachial cutaneous nerves. Despite the presence of these symptoms for the 13 years between the initial fracture and the corrective osteotomy, the patient’s symptoms fully resolved following surgical correction of the malunion (Fig. 5).

## Conclusion

Although ORIF of displaced midshaft clavicle fractures remains controversial in the adolescent population, there may be additional circumstances beyond absolute indications for surgical intervention that warrant ORIF at initial presentation. Potential neurovascular compromises, even if seemingly subtle, should be thoroughly investigated. Careful assessment of the position of the scapula should also be performed and considered in clinical decisions to restore function for the patient. Consideration of more aggressive management of these fractures should be given earlier in the presence of additional symptoms such as parasthesias.

## Disclaimers

Funding: No funding was disclosed by the authors.

Conflicts of interest: The authors, their immediate families, and any research foundation with which they are affiliated have not received any financial payments or other benefits from any commercial entity related to the subject of this article.

Consent: Obtained.
